# Genome sequencing data of extended-spectrum beta-lactamase-producing *Escherichia coli* INF191/17/A isolates of nosocomial infection

**DOI:** 10.1016/j.dib.2022.108407

**Published:** 2022-06-23

**Authors:** Nik Siti Hanifah Nik Ahmad, Khor Bee Yin, Nik Yusnoraini Yusof

**Affiliations:** a School of Health Sciences, Universiti Sains Malaysia, Kubang Kerian, Kelantan 16150, Malaysia; b BioEasy Sdn Bhd, Setia Avenue, 33A-3 Jalan Setia Prima S, U13/S, Setia Alam, Seksyen U13, Shah Alam, Selangor Darul Ehsan 40170, Malaysia; c Institute for Research in Molecular Medicine (INFORMM), Health Campus, Universiti Sains Malaysia, Kubang Kerian, Kelantan 16150, Malaysia

**Keywords:** *Escherichia coli*, Genome sequencing, Extended-spectrum beta-lactamase, Antimicrobial resistant gene

## Abstract

The infection with extended-spectrum beta-lactamase-producing *Escherichia coli* is associated with higher mortality, longer length of hospital-stay and increased costs compared to infection with antibiotic-susceptible E. coli. Here, the draft genome of ESBL-producing E. coli circulating at local hospital is reported. The strain was detected as containing the genes of antibiotic resistance TEM, CTX-M-1, and CTX-M-9. The 5,136,548-bp genome, with a GC content of 50.59%, comprised 4987 protein-coding genes, four ribosomal RNA, and 66 transfer RNA. The ResFinder was successfully predicted fourteen antimicrobial genes in the *E. coli* INF191/17/A genome. Sequence data has been deposited in the GenBank database under the accession number JAIEXV000000000. The BioProject ID in the GenBank database is PRJNA752944. The raw data was sequenced using Ilumina MiSeq and submitted to the NCBI SRA database (SRX11797310), which is publicly available.

## Specifications Table

**Table:** 

Subject	Health and medical sciences
Specific subject area	Microbiology and genomics.Genome sequencing of pathogenic bacteria by using next generation sequencing approach.
Type of data	TableSequencing raw reads in FASTQ format text fileAssembled draft genome of *E. coli* strain INF191/17/A in FASTA format text fileGenome sequence data in FASTA and FASTQ format
How data were acquired	The Illumina MiSeq platform was used to generate paired-end reads of extended spectrum beta lactamase (ESBL)-producing *E. coli* strain INF191/17/A genome.
Data format	Raw data in FASTQ formatAssembled data in FASTA format: GenBank assembly accession: GCA_019599325.1 (https://www.ncbi.nlm.nih.gov/assembly/ GCA_019599325.1).
Parameters for data collection	Bacterial genomic DNA was extracted from a pure culture of ESBL-producing *E. coli* INF191/17/A . Nextera XT DNA library preparation kit was used for the whole-genome sequencing library preparation to generate 2 × 251 paired end reads data.
Description of data collection	Whole genome sequencing was performed using Illumina MiSeq system (Illumina®,USA). BBDuk (BBTools v36) was used to trim raw reads, and SPAdes v3.9.0 was used to assemble clean reads. Genome scaffolding was performed with Medusa v1.6. ResFinder software predicted the putative antimicrobial resistant genes.
Data source location	Institution: Institute for Research in Molecular Medicine (INFORMM)City/Town/Region: Kubang Kerian, KelantanCountry: MalaysiaLatitude and longitude for collected samples/data: 6.10 N 102.28 E
Data accessibility	The data is hosted on a public repository.Bioproject: https://www.ncbi.nlm.nih.gov/bioproject/PRJNA752944Biosample: https://www.ncbi.nlm.nih.gov/biosample/SAMN20668118NCBI GenBank Accession Number: JAIEXV000000000https://www.ncbi.nlm.nih.gov/nuccore/JAIEXV000000000Repository name: NCBI SRA databaseData identification number: SRR15497613Direct URL to data: https://trace.ncbi.nlm.nih.gov/Traces/sra/?run=SRR15497613

## Value of the Data

• The whole genome sequencing data provides insight into genomic determinants of the ESBL-producing *E. coli* strains INF191/17/A and antimicrobial resistance (AMR) genes.
• This data should be used by researchers and public health officers to keep up surveillance and control of ESBL-producing gram negative organisms in order to prevent the emergence of highly resistant strain*,* which is one of serious problem in the world.
• The genome data of *E. coli* strain INF191/17/A accelerates knowledge for pathogenic microbial research in the context of comparative studies, pan-genome, and evolution of non-ESBL and ESBL strains within different epidemiology.
• Furthermore, prior to biomarker discovery, drug or vaccine development, the comprehensive understanding of the whole genome of this pathogen is critically important.

## 1 Data Description

The *Escherichia coli* INF191/17/A was discovered as an extended-spectrum beta-lactamase (ESBL) strain carrying the antibiotic resistance genes TEM, CTX-M-1, and CTX-M-9 via polymerase chain reaction using ESBL specific primers [1]. The 251 base-pair paired-end (2 × 251 bp) sequencing raw reads of the *E. coli* strain INF191/17/A genome were obtained from the Illumina MiSeq system (Illumina, CA, USA) [2]. The raw reads were pre-processed before the genome assembly and annotation. Antimicrobial resistant genes were predicted using curated public database. Genomic DNA was extracted from *E. coli* strain INF191/17/A and sequenced to generate a total of 1,368,224 reads in a 500-cycle run. The total reads from a paired-end dataset (191-17-A_R1.fastq and 191-17-A_R2.fastq) have resulted in 329,238,355 total bases (Table 1). The pre-processed of raw reads including trimming adapter sequences, low-quality and short reads, resulting 46.9% of clean readings. *De novo* assembly of the clean reads was performed and generated 314 contigs with a total size of 5.12 Mbp. Scaffolding resulted in 74 scaffolds with the longest scaffold is 2,520,446 and N50 scaffold length of 1,733,129 bases (Table 2). The average coverage of assembled sequence is 66x with 50.59% of G+C content. Using PGAP, a total of 4987 coding sequences (CDS), four ribosomal RNA, and 66 transfer RNA (Table 3) were predicted. Furthermore, ResFinder predicted that *E. coli* INF191/17/A will develop fourteen antibiotic resistance genes (Table 4).

**Table 1:** Statistics of the raw and clean reads data including forward (191-17-A_R1.fastq) and reverse (191-17-A_R2.fastq) reads.

191-17-A	R1	R2	Total
Total Raw Reads	684,112	684,112	1,368,224
Total Raw Reads Bases	164,465,730	164,772,625	329,238,355
Total Clean Reads	320,871	320,871	641,742
Total Clean Reads Bases	54,470,383	40,781,248	95,251,631
Clean Reads (%)	46.90	46.90	46.90

**Table 2:** The statistics of the assembled draft genome of *E. coli* strain INF191/17/A.

Attributes	Value
Number of scaffolds	74
Total size of scaffolds	5,136,548
Longest scaffold	2,520,446
Shortest scaffold	204
Number of scaffolds > 1 K nt	51 (68.9%)
Number of scaffolds > 10 K nt	21 (28.4%)
Number of scaffolds > 100 K nt	3 (4.1%)
Number of scaffolds > 1 M nt	2 (2.7%)
Number of scaffolds > 10 M nt	0 (0.0%)
Mean scaffold size	69,413
Median scaffold size	2736
N50 scaffold length	1,733,129
L50 scaffold count	2

**Table 3:** The annotation of draft genome of *E. coli* INF191/17/A.

Attributes	Value
Total number of genes	5062
Number of coding sequences	4987
Number of genes (coding)	4736
Total number of RNAs	75
Number of rRNAs	4
Number of tRNAs	66
Number of ncRNAs	5
Number of pseudogenes	251

**Table 4:** Antimicrobial resistance genes and their corresponding antibiotics detected in the *E. coli* INF191/17/A.

AMR gene	Description	Resistance
*mdf(A)*	Multidrug transporter MdfA	Fluoroquinolone, Aminoglycoside, Tetracycline, Macrolide, Rifamycin, Phenicol
*aph(3′')-Ib*	Aminoglycoside resistance protein B	Streptomycin
*aac(3)-IId*	Aminoglycoside-(3)-N-acetyl-transferase (aacC2) gene	Apramycin, Gentamicin, Tobramycin, Dibekacin, Netilmicin, Sisomicin
*aph(6)-Id*	Inosamine-phosphate amidinotransferase	Streptomycin
*aadA5*	Streptomycin and spectinomycin resistance aminoglycoside adenyltransferase	Spectinomycin, Streptomycin
*tet(A)*	Trimethoprim resistant dihydrofolate reductase	Doxycycline, Tetracycline
*mph(A)*	Macrolide 2′-phosphotransferase I	Erythromycin, Azithromycin, Spiramycin, Telithromycin
*sitABCD*	Periplasmic binding protein (sitA), ATP-binding component (sitB), inner membrane component (sitC), inner membrane component (sitD)	Hydrogen peroxide
*blaTEM-1B*	Bet-lactamase TEM-1	Amoxicillin, Ampicillin, Cephalothin, Piperacillin, Ticarcillin
*blaCTX-M-27*	Beta-lactamase CTX-M-27	Amoxicillin, Ampicillin, Aztreonam, Cefepime, Cefotaxime, Ceftazidime, Ceftriaxone, Piperacillin, Ticarcillin
*sul2*	Dihydropteroate synthase type-2	Sulfamethoxazole
*sul1*	Dihydropteroate synthase type-1	Sulfamethoxazole
*dfrA17*	Dihydrofolate reductase	Trimethoprim
*qacE*	Quaternary ammonium compound-resistance protein QacE	Benzylkonium chloride, Ethidium bromide, Chlorhexidine, Cetylpyridinium chloride

## 2 Experimental Design, Materials and Methods

### 2.1 Sample Collection and Isolation of ESBL *E. coli* Strain INF191/17/A

*E. coli* strain INF191/17/A was isolated from a 45-year-old male patient who was suffering from a high fever at a local hospital. In brief, the sample was cultured in the Bactec 9240 blood culture system (Becton, Dickinson, USA) before proceeding with the biochemical testing and gram staining [3]. The ESBL screening and disk confirmation tests were measured according to Clinical and Laboratory Standards Institute (CLSI) [4]. The 16S rRNA sequences for this strain were validated using specific primers of *E. coli* [5]. Then, the PCR was conducted using ESBL-primers for the confirmation of ESBL-type [1].

### 2.2 DNA Isolation, Genome Sequencing, Assembly, and Annotation

Genomic DNA was isolated using NucleoSpin tissue DNA, RNA, and protein purification kit according to manufacturer's instructions (Macherey-Nagel). The purified DNA was processed using Nextera XT DNA library preparation kit following the manufacturer's instructions (Illumina, USA). A whole-genome sequence was performed using the Miseq platform (Illumina, USA) (2 × 251 bp). The adapter trimming, quality trimming, contaminant filtering and read length filtering were performed using BBDuk (BBTools version 36) (http://jgi.doe.gov/data-and-tools/bbtools/). The low-quality bases (<Q30) and short reads (<50 bp) were trimmed to produce clean reads with a high quality read dataset. The clean reads were assembled *de novo* using SPAdes v3.9.0 [6] to obtain contigs. These assembled contigs were subjected to scaffolding against the closest reference genomes [3] to produce a draft genome using Medusa (Multi-Draft based Scaffolder) software [7]. The genome was annotated using the NCBI Prokaryotic Genome Annotation Pipeline (PGAP) v4.10 [8].

### 2.3 Antimicrobial Resistant Genes Analysis

ResFinder (v4.1) [9] was used to screen for antimicrobial resistance genes. The assembled genome was searched against the curated *Escherichia coli* database using the default parameters. The prediction of the genes was confirmed if the assembled sequence had at least 95% nucleotide matching identity and 80% coverage with candidate genes in the database.

## Ethics Statement

The study protocol was approved by the ethics committee of the Universiti Sains Malaysia (USM/JEPeM/20030152).

## CRediT authorship contribution statement

**Nik Siti Hanifah Nik Ahmad:** Software, Formal analysis, Writing – review & editing, Funding acquisition. **Khor Bee Yin:** Conceptualization, Software, Formal analysis, Data curation, Writing – original draft. **Nik Yusnoraini Yusof:** Conceptualization, Software, Methodology, Resources, Writing – review & editing, Supervision, Funding acquisition.

## Declaration of Competing Interest

The authors declare that they have no known competing financial interests or personal relationships which have, or could be perceived to have, influenced the work reported in this article.
